# Does self-management for return to work increase the effectiveness of vocational rehabilitation for chronic compensated musculoskeletal disorders? - Protocol for a randomised controlled trial

**DOI:** 10.1186/1471-2474-11-115

**Published:** 2010-06-10

**Authors:** Niki Ellis, Venerina Johnston, Susan Gargett, Alison MacKenzie, Jennifer Strong, Malcolm Battersby, Rebecca McLeod, Keith Adam, Gwendolen Jull

**Affiliations:** 1 Institute for Safety, Compensation and Recovery Research, Monash University, 499 St Kilda Road, Melbourne, Victoria, 3004, Australia; 2 NHMRC Centre for Clinical Research Excellence - Spinal Pain, Injury and Health, School of Health and Rehabilitation Sciences, The University of Queensland, Chancellors Place, St Lucia, Brisbane, Queensland, 4072, Australia; 3 Centre of National Research on Disability and Rehabilitation Medicine (CONROD) School of Medicine, The University of Queensland, Mayne Medical School, Herston Road, Herston, Brisbane, Queensland, 4006, Australia; 4 Centre for Military and Veterans' Health, The University of Queensland, Mayne Medical School, Herston Road, Herston, Brisbane, Queensland, 4006, Australia; 5 School of Health and Rehabilitation Sciences, The University of Queensland, Chancellors Place, St Lucia, Brisbane, Queensland, 4072, Australia; 6 Flinders Human Behaviour and Health Research Unit, Department of Psychiatry, Flinders University, Margaret Tobin Centre, Flinders Medical Centre, Bedford Park, Adelaide, South Australia, 5001, Australia; 7 Medibank Health Solutions, 340 Adelaide Street, Brisbane, Queensland, 4000, Australia

## Abstract

**Background:**

Musculoskeletal disorders are common and costly disorders to workers compensation and motor accident insurance systems and are a leading contributor to the burden of ill-health. In Australia, vocational rehabilitation is provided to workers to assist them to stay in, or return to work. Self-management training may be an innovative addition to improve health and employment outcomes from vocational rehabilitation.

**Methods/Design:**

The research plan contains mixed methodology consisting of a single blind randomised controlled trial, an economic evaluation and qualitative research. Participants (n = 366) are volunteers with compensated musculoskeletal disorders of 3 months to 3 years in duration who were working at the time of the injury/onset of the chronic disorder. The trial tests the effectiveness of usual vocational rehabilitation plus the Chronic Disease Self-Management Program (CDSMP) to which two additional and newly-developed modules have been added, against vocational rehabilitation alone (control) The modules added to the CDSMP focus on how to navigate through compensation systems and manage the return to work process, and aim to be relevant to those in a vocational rehabilitation setting.

The primary outcome of this study is readiness for return to work which will be evaluated using the Readiness for Return-to-Work scale. Secondary outcomes include return to work status, health efficacy (heiQ™ questionnaire) and general health status (SF-12v2^® ^Health Survey). Measures will be taken at baseline, immediately post-intervention and at 6- and 12- months post-intervention by an independent assessor. An economic evaluation will compare the costs and outcomes between the intervention and control groups in terms of cost-effectiveness and a partial cost-benefit or cost analysis. The impact of the intervention will also be evaluated qualitatively, in terms of its acceptability to stakeholders.

**Discussion:**

This article describes the protocol for a single blind randomised controlled trial with a one year follow-up. The results will provide evidence for the addition or not of self-management training within vocational rehabilitation for chronic compensated musculoskeletal disorders.

**Trial Registration:**

Australia and New Zealand Clinical Trials Registry ACTRN12609000843257

## Background

Arthritis and musculoskeletal conditions are responsible for the third largest proportion of health expenditure and are a national health priority for Australia [1]. They are responsible for over 50% of claims in Australian workers compensation schemes [2]. There is a strong evidence-base for early return to work as a part of the management of these conditions [3]. The International Labour Organisation defines vocational rehabilitation as activities which enable a disabled person to secure and retain suitable employment [4]. This includes medical, psychological, social and occupational activities which aim to re-establish the working capacity of sick or injured persons with a previous work history as well as provide the prerequisites for returning to the labour market [5]. Thus, vocational rehabilitation incorporates work re-training, education and counselling, work guidance, ergonomic modifications and psychosocial strategies.

Vocational rehabilitation has grown significantly in Australia since the 1980s in response to rapidly rising workers' compensation costs and following the introduction of vocational rehabilitation into workers compensation legislation [6]. The rationale underpinning its availability is cost savings for communities and employers and the improved health of individuals through early return to work. However, vocational rehabilitation does not have a substantial evidence-base to support its effectiveness [7], and so has come under question especially with the rising cost of the growing vocational rehabilitation industry [8-10]. In addition, a recent review [11] criticised vocational rehabilitation for coming too much under the influence of economic rationalism and called for a return to its humanitarian core. The review proposed that ideas being put forward in relation to health reform, including self-management, could assist with this aim.

Self-management is an approach used increasingly in chronic illness care to improve self-efficacy and wellness behaviours [12]. It has been defined as the learning and practising of the skills necessary to conduct an active and emotionally satisfying life while living with a chronic condition [13]. Self-management programs aim to help participants make informed choices and then carry them out [13]. Key self-management skills include; problem solving, decision making, resource utilization, forming partnerships with healthcare providers and taking action [14]. Program participants are up-skilled in personalised goal setting and action care planning, and collaborative problem definition is based on their readiness to change and self-efficacy. Chronic disease self-management programs have been shown to improve clinical outcomes and health care utilisation in some chronic diseases [12,15].

This research will investigate the benefit or not of adding self-management to usual vocational rehabilitation for injured persons who have transitioned from the acute to chronic stage of a musculoskeletal condition. The Chronic Disease Self-Management Program (CDSMP), developed at the Stanford University [16], was chosen for use in this research as it was judged to be the most appropriate for the study population. For the purposes of this research, two new modules have been developed and added to the CDSMP to tailor the self-management intervention to those participating in vocational rehabilitation and seeking to return to work post-injury.

Three hypotheses drive this research: first, that a greater proportion of individuals who participate in the self-management program will be classified as ready to return to work based on a validated scale; second, that adding self-management to vocational rehabilitation will result in a more efficient use of resources and third, that the self-management intervention will be acceptable to vocational rehabilitation clients, health care providers, policy makers and regulators.

The aims of this research are:

1. To develop an intervention to add self-management to vocational rehabilitation for individuals with chronic compensated musculoskeletal disorders.

2. To test the effectiveness of self-management plus usual vocational rehabilitation against usual vocational rehabilitation alone.

3. To undertake an economic evaluation of adding self-management to usual vocational rehabilitation practice.

4. To determine the acceptability of the intervention to clients, their vocational rehabilitation providers, policy makers and regulators.

## Methods/Design

The methodology below was developed by the scientific research team in consultation with the industry partners.

### Design

The design is mixed methods research consisting of a single blind randomised controlled trial, an economic evaluation and qualitative research. The trial will test an intervention of self- management training plus usual vocational rehabilitation against usual vocational rehabilitation care only. Ethical approval has been granted from the Human Research Ethics Committee of The University of Queensland (2009000579). Informed written consent will be obtained from all participants.

### Participants

Participants will comprise eligible volunteers who are clients either referred to a national provider of occupational health services and vocational rehabilitation or referred directly to the trial by their insurer. Recruitment will be undertaken in two Australian cities. To be eligible for the study volunteers will have: a diagnosed, compensated musculoskeletal disorder of 3 months or more but not longer than 3 years in duration; been working at the time of the injury/onset of the chronic disorder; and English language and literacy skills that are adequate to complete the study requirements including participating in the self-management course. In addition, there must be written agreement by the payer (insurer) for the individual to participate. Individuals will not be eligible if they have signs and symptoms suggestive of non musculoskeletal conditions (e.g. tumour, systemic illness, inflammatory disease or infection), their primary condition is a psychological/psychiatric or neurological disorder, or if they have previously participated in a vocational rehabilitation, chronic pain, functional restoration or work hardening program as these may contain a self-management component.

The sample size calculation was based on the primary outcome readiness for return to work status at 12 months post-intervention. A power calculation was performed assuming that the proportion ready to return to work in the usual care group would be 30% and the proportion in the self-management group would be 45%. Accounting for a 10% loss to follow-up, a total of 183 participants were required in each study arm (total n = 366) for the study to achieve 80% power.

### Randomisation

Volunteers accepted into the study will be randomised into an intervention or control group by an independent body using a randomised permuted block design. Prior to randomisation, participants will be stratified by state to cater for local regulations and conditions. The scientific research team are blinded to the randomisation process and all data collection.

### Intervention

The control group will receive usual vocational rehabilitation only. The intervention group will receive vocational rehabilitation plus the self-management intervention which has been termed Self-Management for Return to Work (SMRTW). It comprises eight, two-hour weekly sessions specifically, the six modules of the CDSMP plus two new modules relevant to a vocational rehabilitation setting. The SMRTW program will be conducted for groups of 8-10 participants. The new modules were developed to compliment the format and approach of the CDSMP. In order to be classified as having received the intervention, participants must attend at least five of the eight sessions including at least one of the new module sessions.

In developing the two additional modules, the research team consulted industry partners including a national leader in occupational and vocational rehabilitation and experts in self-management training. The (new) module titled *Navigating the System *aims to: assist the client understand the agency funding their rehabilitation, its function and services; encourage them to use problem-solving skills to deal with 'system' issues; and, enhance their knowledge of the various persons in the 'system' and their respective roles. The (new) module titled *Managing Return-to-Work *aims to assist the client to understand: their role in the return to work process; the factors enhancing and limiting the success of return to work; and, the implications of having people with injuries in the workplace for employers and co-workers. As this is the final module delivered in the program, participants are also encouraged to reflect on the skills they have learnt during the program and to plan for their futures.

### Outcome measurements

Measurements will be taken at baseline, immediately post-intervention (i.e., within 7 days of completion of the intervention) and at 6-month and 12-month follow-up points. All follow-up assessments will be conducted by a researcher blinded to the participant's group allocation. The researcher conducting the data entry process will be blinded to group allocation.

#### Baseline data

Socio-demographic data will be collected at baseline including the participant's age, gender, martial status, educational attainment, current work status, employment history (i.e. industry currently/previously employed in), insurance type, the nature of the musculoskeletal injury/condition and the time since their injury.

#### Primary outcome measure

Readiness for return to work: This will be evaluated using the Readiness for Return-to-Work (RRTW) scale [17], a 22-item scale which applies the readiness for change model to identify individual and social factors impacting on an individual's ability to initiate and maintain behaviour change i.e. return to work after an injury. The psychometric properties of the scale have been evaluated and found to be acceptable [17]. Six dimensions have been identified of which four are relevant for those not working and two for those working. The non-working dimensions are: Pre-contemplation; Contemplation; Prepared for action - behavioral; and, Prepared for action - self evaluative. The two dimensions for those working are Uncertain maintenance and Proactive maintenance. The mean value for each dimension is calculated with a higher score in a particular stage indicative of the level the individual is at. For the purposes of this study, readiness for return to work will be determined by higher scores in the Prepared for action - self evaluative, Uncertain maintenance, and Proactive maintenance dimensions. The latter two stages were included to ensure that those working were not excluded on the basis of having returned to work. Those in the Prepared for action - self evaluative stage will be classified as ready to return to work to avoid misclassifying those who are found to be ready for return to work but have been unable to obtain employment.

#### Secondary outcome measures

Secondary outcomes include return to work status, health efficacy, and general health status.

• Return to work status will be documented.

• The Health Education Impact Questionnaire (heiQ™) is a reliable measure with high construct validity, designed to evaluate outcomes from patient education and self-management interventions for people with chronic conditions. It comprises eight domains to assess more proximal program outcomes [18].

• The SF-12v2^® ^Health Survey will be used to measure health status. It consists of 12 items from the widely-used SF-36 Health Survey and provides summary assessment measures of physical and mental health [19].

#### Economic outcome measures

The relative efficiency of adding the self-management intervention to usual vocational rehabilitation care will be assessed through cost and outcome data. Incremental cost-effectiveness ratios (ICERs) will be calculated in relation to the four outcome measures and will be estimates of the extra cost from adding the SMRTW intervention per extra unit of outcome/effect, e.g. the extra cost per extra participant classified as ready to return to work or per extra participant who returned to work. That is;(1)

where the subscripts '*I*' and '*C*' indicate the intervention and control groups, respectively. A partial cost-benefit (or cost) analysis will assess the net benefit, or cost, from adding the intervention. This measure is the difference (in dollars) in the benefits and costs between those who received the intervention and those who did not. Specifically;(3)

This analysis is referred to as a partial cost-benefit analysis (or cost analysis) as the benefit of the SMRTW program will be measured only in relation to participants' production of paid and unpaid work. Other potential benefits of the program such as gains in health status or health efficacy will not be estimated or included, and willingness-to-pay for the intervention will not be determined.

### Procedures

#### Preparation of Facilitators

The SMRTW program will be facilitated by two people: a lay facilitator trained in the self-management model and a vocational rehabilitation practitioner. The practitioners (n = 5) are employees of one of the project's industry partners, a national provider of vocational rehabilitation services. They will undertake an accredited four day train-the-trainer training program in self-management conducted by Arthritis Queensland.

**Figure 1:**
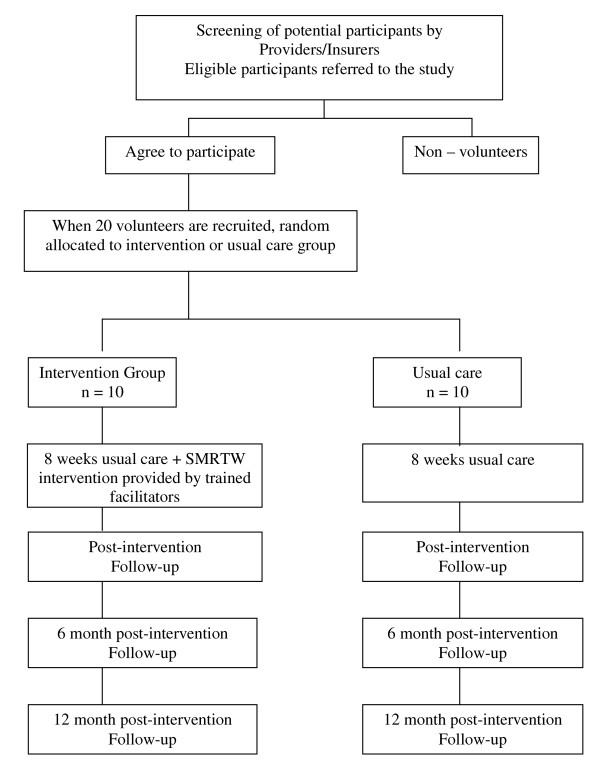
**Flow chart of the research protocol**.

A flow chart of the trial is presented in Figure 1. The recruitment period will be 18 months. Participants will be notified by the project manager via telephone when sufficient participants are recruited to constitute an intervention, and control group. This notification will occur up to two weeks prior to the commencement of an eight-week intervention period. The self-management sessions will be conducted at the offices of the vocational rehabilitation provider involved in the research. Outcome data will be collected via a telephone interview. In addition, to assess the uptake of the intervention, recruiting organisations will be asked to record the number of non-volunteers and their reasons for not participating.

### Data Analysis

Analysis of the effectiveness of the SMRTW program will be performed on an intention to treat basis and per protocol. Primary outcomes of interest are those collected at 12 months post-intervention. The differences in the mean scores of the continuous variables will be analyzed using multivariate regression and the difference in the proportion of the dichotomous outcomes (e.g. the proportion 'ready to return to work') will be analyzed using logistic regression.

Continuous outcome measures will be analyzed using multi-level modeling to describe within participant differences over time and to account for the repeated measures design component of the study. In the modeling of continuous outcomes, the baseline measures collected pre-intervention will be included as covariates in the model. Based on the results observed, participant, time and intervention will be defined as fixed or random effects as appropriate. Similarly, multivariate logistic regression will be used to assess dichotomous (1/0) outcomes. The effect of the SMRTW program on the outcomes at the earlier time points (i.e. immediately and 6 months post-intervention) will also be analyzed after appropriate adjustments to the significance level to account for multiple testing.

In regards to the economic evaluations, service utilisation, cost and work outcome data will be obtained from the questionnaires administered to participants at the four data collection time points. Estimates of these measures for the intervening time periods shall be determined by linear interpolation. Summary data on the measures will be presented and compared statistically for the two groups.

To derive the ICERs, the cost-effectiveness analyses will be performed from the perspective of payers e.g. insurers and/or government. Estimated costs to these funders will include their expenditures on income/disability benefits, and vocational rehabilitation and health care services. Utilisation data for vocational rehabilitation and formal (e.g. general practitioner attendances and medication use) and informal health or social care services (e.g. community services) will be used to estimate the costs. In contrast, the partial cost-benefit (or cost) analysis will adopt a societal perspective in relation to costs. Thus, costs to participants and their families (e.g. time and travel costs) shall be estimated and added to the estimates of costs to payers. Sensitivity analyses may be conducted to allow for uncertainty in key assumptions or variables.

### Qualitative analysis

The impact of the SMRTW intervention will also be evaluated in terms of its acceptability to stakeholders through focus group discussions. These will be undertaken to explore the perceptions, experiences and understandings of various stakeholder groups (specifically, participants, practitioners, insurers, and project partners) regarding the impact and acceptability of the program. The groups will consist of 8 to 10 representatives from each group and the analysis will be conducted in the final year of the study. This qualitative method is well suited to such research regarding health and health care [20].

Additionally, an audit of 40 provider case files (20 treatment and 20 controls) will be undertaken at the mid point of the study. The files will be audited to gauge the extent to which the practice of self-management may be impacting on the 'usual care' of the provider.

## Discussion

Significant decisions made by this project team in developing this methodology are now outlined:

• Whether a generic self-management program would meet the needs of this population was an issue. It was decided it would, with the addition of two new modules described above.

• The project industry partners advised that co-morbidity, especially with psychiatric conditions, was common and an issue of concern to them. It was decided to include people with psychiatric co-morbidity, but to exclude those for whom the primary cause of their claims was a psychiatric condition or a significant non-musculoskeletal condition.

• There were some different views about the best duration of condition to include in the study. Researchers initially favoured acute or sub-acute conditions based on the knowledge that earlier interventions are usually more likely to be acceptable. Industry partners favoured chronic conditions as knowledge about how to deal with these is most lacking. A final compromise was cases of more than 3 months duration (i.e. just chronic) but not more than 3 years.

• Defining what was meant by compensation was not as simple as expected. Researchers were keen to include people on a broad range of compensation schemes, specifically workers compensation, transport accident compensation and disability benefits. The industry partners who were contributing some funds became concerned as it appeared that the study population may be predominantly people on disability benefits given the original recruitment strategy. In response the recruitment strategy was broadened to that described above to redress the imbalance.

• Consideration was given to whether to include return to work status as the primary outcome measure. However, to avoid the ambiguity surrounding the definition of return to work and to ensure we capture a complete picture of the complex return to work process, we decided to employ readiness for return to work as our primary outcome measure [21].

• Defining a scope for the economic evaluation which was feasible within the scope of this project yet valuable was a challenge. Ultimately it was agreed to conduct a cost-effectiveness and partial cost-benefit study as described.

Close collaboration between the researchers and industry partners during the formative stages of the development of the methodology proved to be important in ensuring the research would meet the needs of industry.

This project stands to provide information on the acceptability, effectiveness and efficiency of adding self-management training to vocational rehabilitation for the management of people with chronic compensated musculoskeletal disorders; conditions that are responsible for a high proportion of the total health burden and of costs to governments, workers compensation and motor accident insurers. It also stands to enhance knowledge on the benefits, or otherwise, of broadening settings that are supportive of self-management to include workplaces and rehabilitation services. In addition, the research will add knowledge in the domain of vocational rehabilitation, a field criticised for its lack of evidence to support accepted practice. Such information is necessary if scarce resources are to be used efficiently in the maximisation of health and return to work outcomes. Finally, this research will generate knowledge on empowering people with chronic compensated conditions to play a greater role in managing their disorders within the context of compensation systems and return to work.

## Competing interests

The authors declare that they have no competing interests and no author will receive any reimbursements, fees or salary for performing the study.

## Authors' contributions

NE, GJ, VJ, SG, AM, JS, MB conceived and designed the research plan and wrote the manuscript. KA, RM assisted with the study design and writing of the manuscript. All authors read and approved the final manuscript.

## Pre-publication history

The pre-publication history for this paper can be accessed here:

http://www.biomedcentral.com/1471-2474/11/115/prepub

## References

[B1] Australian Institute of Health and Welfare Arthritis and musculoskeletal conditions in Australia 2005. With a focus on osteoarthritis, rheumatoid arthritis and osteoporosis AIHW CAT. NO. PHE 67 2005

[B2] Australian Safety and Compensation Council Compendium of workers' compensation statistics Australia 2006-07 2009 Canberra: Australian Government

[B3] Accident Compensation Corporation New Zealand Acute Low Back Pain Guide incorporating the guide to assessing psychosocial yellow flags in acute low back pain 2004

[B4] International Labour Organisation Vocational rehabilitation and employment of disabled persons International Labour Conference 86th Session 1998

[B5] Selander J Return to work following vocational rehabilitation for neck, back and shoulder problems: risk factors reviewed Disability and Rehabilitation 2002 24 14 704 712 10.1080/09638280210124284 12396655

[B6] Ellis N Work and Health: Management in Australia and New Zealand 2001 Melbourne: Oxford University Press

[B7] Industry Commission Workers' compensation in Australia 1994

[B8] Smith L Providing better care for patients with chronic disease Australian Journal of Primary Health 2003 9 2-3 119 26

[B9] Underhill E Extending Knowledge on Occupational Health & Safety and Labour Hire Employment: A Literature Review and Analysis of Victorian Worker's Compensation Claims 2002 WorkSafe Victoria

[B10] The Institute for Research into International Competitiveness (IRIC) Cost benefit analysis of rehabilitation services provided by CRS Australia Journal of Contemporary Issues in Business and Government 2003 11 1 71 94

[B11] Kendall E Trends in Australian rehabilitation: Reviving its humanitarian core Disability & Rehabilitation 2007 29 10 817 823 10.1080/09638280600919731 17457740

[B12] Lorig K Mazonson P Holman H Evidence suggesting that health education for self-management in patients with chronic arthritis has sustained health benefits while reducing health care costs Arthritis and Rheumatism 1993 36 4 439 446 10.1002/art.1780360403 8457219

[B13] Lorig K Self-management of chronic illness: a model for the future Generations 1993 17 3 11 14

[B14] Lorig K Holman H Self-management education: history, definition, outcomes, and mechanisms Annals of Behavioral Medicine 2003 26 1 1 7 10.1207/S15324796ABM2601_01 12867348

[B15] Lorig K Holman HR Long-term outcomes of an arthritis self-management study: effects of reinforcement efforts Soc Sci Med 1989 29 2 221 4 10.1016/0277-9536(89)90170-6 2665110

[B16] Stanford School of Medicine and Stanford University Chronic Disease Self Management Program (CDSMP) Stanford Patient Education Research Center http://patienteducation.stanford.edu/programs/cdsmp.html

[B17] Franche RL The Readiness for Return-To-Work (RRTW) scale: Development and Validation of a Self-report Staging Scale in Lost-time Claimants with Musculoskeletal Disorders Journal of Occupational Rehabilitation 2007 17 3 450 472 10.1007/s10926-007-9097-9 17701326

[B18] Osborne RE Elsworth GR Whitfield K The Health Education Impact Questionnaire (heiQ): An outcomes and evaluation measure for patient education and self-management interventions for people with chronic conditions Patient Education and Counseling 2007 66 192 201 10.1016/j.pec.2006.12.002 17320338

[B19] Ware EJ Jr Turner-Bowker KM Gandek-B DM User's Manual for the SF-12v2™ Health Survey with a Supplement Documenting SF-12^® ^Health Survey 2002 Lincoln, RI: QualityMetric Incorporated

[B20] Rice PL Ezzy D Qualitative Research Methods: A Health Focus 1999 South Melbourne: Oxford University Press

[B21] Bültmann U Health status, work limitations, and return-to-work trajectories in injured workers with musculoskeletal disorders Quality of Life Research 2007 16 7 1167 1178 10.1007/s11136-007-9229-x 17616838 PMC2039824

